# Interaction between gold nanoparticles and blood proteins

**DOI:** 10.1080/07853890.2021.1896884

**Published:** 2021-09-28

**Authors:** Carlos Costa, João Cadete, Miguel Peixoto de Almeida, Eulália Pereira, Ludwig Krippahl, Ricardo Franco

**Affiliations:** a UCIBIO, REQUIMTE, Departamento de Química, Faculdade de Ciências e Tecnologia, Universidade NOVA de Lisboa, Caparica, Portugal; b LAQV, REQUIMTE, Departamento de Química e Bioquímica, Faculdade de Ciências, Universidade do Porto, Rua do Campo Alegre, s/n, Porto; c NOVA LINCS, Departamento de Informática, Faculdade de Ciências e Tecnologia, Universidade Nova de Lisboa, Caparica, Portugal

## Abstract

**Introduction:**

Metallic nanoparticles constitute promising biosensing systems, due to their high affinity to biomolecules such as proteins, which form protein coronas of distinct compositions on their surface [1]. Gold nanoparticles (AuNP) are particularly interesting given their relatively easy, quick and inexpensive synthesis, low toxicity and ease of functionalization with bifunctional molecules. Usually, these molecules have thiol groups bound to the AuNP surface and bio-friendly chemical groups at the opposite end, allowing for controlled protein adsorption. Such functionalised AuNPs may be used as probing agents for a patient’s droplet of blood and the health state can be based on the composition of AuNP-adsorbed protein corona. It is thus important to understand the behaviour of each plasma protein in the corona, divided into a tightly-bound inmost monolayer (*hard corona*) and looser outer layers (*soft corona*), removable through centrifugation [2].

**Materials and methods:**

AuNP synthesis was performed according to a modified Turkevich method and AuNP diameter and concentration was determined by UV-Vis spectroscopy. AuNPs were functionalised with 11-mercaptoundecanoic acid and further conjugated with bovine or human serum albumin or fibrinogen. These single protein conjugates were evaluated for hydrodynamic diameter changes after centrifugation by dynamic light scattering (DLS). Agarose gel electrophoresis (AGE) allowed to determine electrophoretic mobility and concentration-dependent conjugation efficiency. Analysis of AGE profiles was by the open source electrophoresis gel image processing software eReuss.

**Results:**

DLS showed a decrease in hydrodynamic diameter for centrifuged conjugates of 40 nm gold nanoparticles, with too high polydispersity indexes for the 13 nm ones, suggesting aggregation. AGE revealed a decrease in electrophoretic mobility as the protein-to-AuNP ratio increases, data fitted to a Langmuir adsorption model. Protein and AuNP concentrations during incubation affect the electrophoretic mobility profile. In fact, depending on the protein-to-AuNP ratio, migration is proportional to the colloidal suspension volume in which conjugation occurred.

**Discussion and conclusions:**

Although centrifugation can induce AuNP aggregation, it appears to affect the protein corona. A decrease in hydrodynamic diameter as determined by DLS appears in centrifuged samples comparatively to its uncentrifuged counterparts, which corroborates the *soft corona* being more loosely bound. Moreover, AGE suggests that for equal protein:AuNP ratios, the volume of sample is determinant for the conjugation process.
